# A Novel Approach to Classification and Reporting of Lymph Node Fine-Needle Cytology: Application of the Proposed Sydney System

**DOI:** 10.3390/diagnostics11081314

**Published:** 2021-07-21

**Authors:** Elena Vigliar, Gennaro Acanfora, Antonino Iaccarino, Massimo Mascolo, Daniela Russo, Giulia Scalia, Roberta Della Pepa, Claudio Bellevicine, Marco Picardi, Giancarlo Troncone

**Affiliations:** 1 Department of Public Health, University of Naples “Federico II”, 80131 Naples, Italy; elena.vigliar@unina.it (E.V.); antiaccc@hotmail.com (A.I.); claudio.bellevicine@unina.it (C.B.); 2 Department of Advanced Biomedical Sciences, University of Naples “Federico II”, 80131 Naples, Italy; gennaro.acanfora95@gmail.com (G.A.); massimo.mascolo@unina.it (M.M.); daniela.russo@unina.it (D.R.); 3 Laboratory of Clinical Research and Advanced Diagnostics, CEINGE Biotecnologie Avanzate, 80131 Naples, Italy; scalia@ceinge.unina.it; 4 Department of Clinical Medicine and Surgery, Hematology Section, University of Naples “Federico II”, 80131 Naples, Italy; robertadellapepa@gmail.com (R.D.P.); marco.picardi@unina.it (M.P.)

**Keywords:** lymph node, fine-needle cytology, reporting system, Sydney system, lymphoproliferative disorders, metastasis

## Abstract

Fine-needle cytology (FNC) is a useful diagnostic tool in the first line evaluation of lymphadenopathy of unknown aetiology. Nevertheless, considering the large number of conditions presenting as lymphadenopathy, lymph node cytology represents a challenging scenario. Recently, an expert panel published the proposal of the Sydney system for performing classification and reporting of lymph node cytopathology; the aim of the present study was to evaluate the applicability of this system. Thus, 300 lymph node FNCs performed over 1 year were reviewed and categorized according to the Sydney system classification. Overall, n = 20 cases (6.7%) were categorized as L1-inadequate/non-diagnostic; n = 104 (34.7%) as benign (L2); n = 25 (8.3%) as atypical (L3); n = 13 (4.3%) as suspicious (L4), and n = 138 (46%) as malignant (L5). FNC diagnoses were correlated with histopathologic and clinical follow-up to assess the diagnostic accuracy and the risk of malignancy (ROM) for each diagnostic category. Statistical analysis showed the following results: sensitivity 98.47%, specificity 95.33%, positive predictive value 96.27%, negative predictive value 98.08%, and accuracy 97.06%. The ROM was 50% for the category L1, 1.92% for L2, 58.3% for L3, and 100% for L4 and L5. In conclusion, FNC coupled with ancillary techniques ensures satisfactory diagnostic accuracy and the implementation of the Sydney system may improve the practice of cytopathologists.

## 1. Introduction

Fine-needle cytology (FNC) is a useful diagnostic tool in the first line evaluation of lymphadenopathy of unknown aetiology. In fact, in addition to the well-known advantages of the cytopathological approach, such as minimum invasiveness, rapidity, and cost effectiveness, the capability to provide material for several ancillary techniques has contributed to improve lymph node FNC accuracy [1]. The diagnosis of malignant lymphadenopaties still relies on excisional biopsy and histological evaluation [2,3]. However, most benign lymphadenopaties may be reliably diagnosed by combining FNC microscopic features with flow cytometry (FC), immunocytochemistry (ICC), microbiological analysis, and molecular testing data [4,5,6,7,8,9,10,11,12,13,14,15,16,17,18,19,20,21,22], thus, avoiding unnecessary diagnostics surgical procedures. Moreover, cytology can be especially useful when a surgery is inadvisable or unfeasible, as in elderly patients with comorbidities or in metastatic settings. 

Nevertheless, lymph node FNC represents a challenging scenario. Considering the large number of benign and malignant conditions presenting as lymphadenopathy, the knowledge of clinical history, physical examination, and radiological/ultrasonographic (USA) features is pivotal for a cytoptahologist as well as the use of a standardized categorization and communication to clinicians [23,24]. To fulfil the latter requirement, in 2020, an expert panel published the proposal of the Sydney system for performing classification and reporting of lymph node cytopathology, introducing the use of five diagnostic categories [25]; moreover, taking into account the wide spectrum of lymph node pathologies, a second diagnostic level, aimed at the identification of specific diagnostic entities, has been proposed. However, the Sydney system is still underutilized and to date there are limited data in the literature [26]. To fill this knowledge gap, the aim of the present study was to evaluate the applicability of the Sydney system to lymph node FNC and to assess the diagnostic accuracy and the risk of malignancy (ROM) for each diagnostic category. 

## 2. Materials and Methods

### 2.1. Study Design 

In this retrospective study, a search of the electronic database of the Cytopathology Division at the University of Naples “Federico II” was carried out focusing on patients who underwent lymph node FNC over 1 year; the period between 1 January 2019 and 31 December 2019 was selected to ensure a clinical follow-up of at least 16 months. All information regarding human material was managed using anonymous numerical codes, and all samples were handled in compliance with the Helsinki Declaration. Pathology records were retrieved and data on patients age, sex, lymph node location, clinical history, ancillary studies and final diagnosis were recorded.

### 2.2. Cytological Samples

In all cases, FNCs were performed by an experienced cytopathologist under US control; in cases in which a lymphoproliferative disorder was suspected or in deep-located lymph nodes, FNCs were assisted by a hematologist with more than 15 years of experience with interventionist power-doppler US. The diagnostic procedure and its related risks were first discussed with the patients and informed consent was obtained. A 23-gauge needle was used, and the first pass served to prepare a direct smear, on-site Diff–Quik stained and microscopically evaluated for the adequacy assessment and the specimen triage. In those cases showing uncertain microscopy, with overlapping features between small cell non-Hodgkin lymphomas (NHL) and reactive lymphadenopathies, the remaining material present in the hub of the needle was flushed out with phosphate-buffered saline solution (PBS) for FC analysis; in cases where the differential diagnosis included large cell NHL, Hodgkin lymphoma (HL), or metastases, residual material in the needle was suspended in 5 mL of 10% neutral buffered formalin, for ICC on cell block (CB) preparation. A second pass was performed in cases yielding scant cellularity. FC analysis and CB preparation were carried out, as previously described [15].

### 2.3. Diagnostic Categories

The original diagnoses were reviewed, and each case was assessed according to the first diagnostic level of the Sydney system classification (L1, inadequate/nondiagnostic; L2, benign; L3, atypical cells of undetermined significance/atypical lymphoid cells of uncertain significance (AUS/ALUS); L4, suspicious; L5, malignant). Any discrepancies in the classification were resolved by consensus between at least two pathologists. The second diagnostic level, when feasible, was recorded. 

### 2.4. Histopathologic Correlation and Clinical Follow-Up

To assess the diagnostic accuracy and the ROM for each diagnostic category, histopathologic diagnoses were correlated with FNC diagnoses; when no biopsy was performed, clinical follow-up was checked. 

### 2.5. Statistical Analysis

The sensitivity, specificity, positive predictive value (PPV), negative predictive value (NPV), and overall diagnostic accuracy of lymph node FNC were assessed. To this end, a true positive was defined as any histologically or clinically confirmed malignant lesion with a malignant (L5), suspicious (L4) or atypical cytological diagnosis (L3); a true negative was defined as any histologically or clinically confirmed benign lesion with a benign (L2) diagnosis; a false positive was defined as any histologically benign lesion with an L5, L4, or L3 cytological diagnosis; a false negative was defined as any histologically malignant lesion with an L2 cytological diagnosis. FNC samples yielding inadequate/nondiagnostic material (L1) were excluded from these analyses. 

ROM was calculated by dividing the number of cases with a confirmed malignant lesion by the total number of cases with a histological or clinical follow-up within each diagnostic category. 

## 3. Results

### 3.1. Cytological Samples

Overall, 300 lymph node FNCs were performed from patients of all ages, ranging from 13 to 85 years (mean age 54.6 y) and both sexes (n = 173 females (57.7%) and n = 127 men (42.3%)). The lymph node locations included cervical group (n = 136, 45.3%), axillary (n = 55, 18.3%), mandibular (n = 40, 13.3%), inguinal (n = 29, 9.7%), supraclavicular (n = 26, 8.7%), abdominal (n = 8, 2.7%), pectoral (n = 2, 0.7%), iliac obturator (n = 1, 0.3%), pararectal (n = 1, 0.3%), tracheal (n = 1, 0.3%), and sternal (n = 1, 0.3%); lymph node size ranged from 9 to 72 mm (mean size 24 mm). In 66 cases (22%) FNCs were performed on patients with a history of a previous diagnosis of malignancy (n = 30 lymphoma and n = 36 carcinoma). In 179 of 300 cases, ancillary techniques were required, in particular, n = 109 (36.6%) ICC analysis, n = 84 (28%) FC analysis, and n = 1 (0.3%) molecular testing were performed.

### 3.2. Diagnostic Categories

In the present series, n = 20/300 (6.7%) were re-categorized as L1, inadequate/non-diagnostic; n = 104/300 (34.7%) as L2, benign; n = 25/300 (8.3%) as L3, AUS/ALUS; n = 13/300 (4.3%) as L4, suspicious, including n = 6 suspicious for NHL, n = 3 suspicious for HL and n = 4 for metastasis. Finally, the majority of cases were categorized as L5, malignant (n = 138, 46%) further classified into NHL (n = 48), HL (n = 9) and metastasis (n = 81). The high proportion of malignant diagnoses is presumably related to the fact that, as an academic hospital, we represent a referral center for selected patients. Sample characteristics, clinical data, and diagnostic categories are summarized in Table 1. The second diagnostic level was provided in 115 cases (38.3%). Data regarding the distribution of the second diagnostic level are summarized in Table 2.

### 3.3. Histopathologic Correlation and Clinical Follow-Up

**Figure 1:**
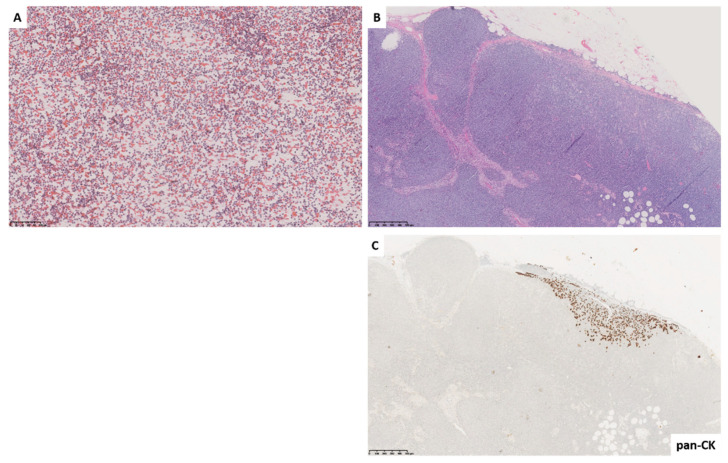
Fine needle cytology smear shows a dispersed polymorphous cell population represented by small lymphocytes and rare medium-sized follicle center cells; no epithelial groups were observed ((**A**) Papanicolaou stain). Histological control shows partial, subcapsular involvement of the lymph node by atypical, dischoesive epithelial cells (pan-cytocheratine positive) ((**B**) hematoxylin and eosin stain and (**C**) pan-cytokeratin AE1/AE3).

Histopathologic correlation was available in n = 103 (34.3%) cases, mostly for L5 diagnostic category; in fact, in 60 cases, histology confirmed malignant cytological diagnoses. Conversely, in the L2 diagnostic category, only n = 20 histopathologic controls were available; of these, two cases proved to be false negative diagnoses as histology revealed the presence of subcapsular breast metastases (Figure 1). As far as the L3 category is concerned, histopathologic correlation was available in 12 cases, five of which showed a benign reactive hyperplasia (BRH); therefore, five false positive diagnoses were recorded in the L3 category. Instead, in n = 7 L4 cases, histology confirmed the cytological diagnosis. Finally, n = 4 histologic controls were available in L1 cases.

N = 139 (46.3%) cases were checked and confirmed clinically by follow-up, including both L2 (n = 84) and L4-L5 (n = 55) diagnosis, that did not undergo surgery due to co-morbidity, disease relapse, or advanced stage disease (n = 14 NHL, n = 41 metastases). Finally, n = 58 cases (19.3%) were lost during the follow-up. Data are summarized in Table 3.

As far as the second diagnostic level is concerned, histopathologic correlation was available and confirmed cytological diagnoses in n = 80/115 samples.

### 3.4. Statistical Analysis

In the present series, statistical analysis showed the following results: sensitivity 98.47%, specificity 95.33%, PPV 96.27%, NPV 98.08%, and accuracy 97.06% (Table 4).

The ROM was calculated for each diagnostic category, when histopathologic correlation or clinical follow-up were available: category L4 and L5 had the higher ROM (100%); the lower value of ROM (1.92%) was observed in category L2. Instead, intermediate ROM values were associated with categories L1 (50%) and L3 (58.3%) (Table 5).

## 4. Discussion

Cytological evaluation of lymphadenopaties can be extremely challenging; nonetheless, a growing body of data show that the proper handling of diagnostic material to perform ancillary techniques, coupled with clinical data, ensures satisfactory diagnostic accuracy; indeed, as reported above, in the present study we demonstrate high diagnostic accuracy. However, the use of lymph nodes FNC is still not uniformly accepted by clinicians, mainly as a consequence of a lack of guidelines and reporting system. As experienced in other fields of cytopathology, the application of standardized reporting systems enables to limit interobserver variability and to communicate clinically relevant information in a reproducible manner [23,24]. Moreover, the rate of clinician misinterpretation of cytological reports might be reduced by using management recommendations, specific for each diagnostic category; to this end, it is crucial to perform risk stratification and to identify ROM values common to several entities.

In the present series, we showed the ability of the Sydney system to stratify lymph node FNCs into categories with increasing ROMs. Interestingly, ROM of L1 category was remarkably high (50%); however, this extremely high value was probably due to the small number of histological controls available (4/20), including two benign lymphadenopathy, one NHL, and one HL. Noteworthy in our series, out of 20 L1 cases, FNCs were performed in two patients on subcentimetric lymph nodes and in eight patients on deep-located or difficult to sample lymph nodes (three abdominal, three axillary, and two supraclavicular). In all these cases, despite performing rapid on-site evaluation (ROSE), material was scant and non-diagnostic, thus, a repetition was inadvisable. Therefore, our experience is consistent with Sydney system management recommendations in the L1 category that include, other than FNC repetition, core-needle biopsy or excision biopsy, based on the specific clinical context [25]. ROSE by an experienced on-site cytopathologist is needed, however, in some institution the lack of personnel and logistic issues may represent a limitation; therefore, the use of advanced methods to improve the diagnostic accuracy of cytopathology, such as liquid-based cytology, should be considered.

As expected, the L2 category showed the lowest ROM (1.92%). Notably, in our series, few infective lymphadenopaties were present; however, the utility of FNC as a non-invasive procedure in this setting in most parts of the world cannot be overemphasized [26]. Interestingly, the two FN diagnoses were represented by subcapsular breast metastases in axillary lymph nodes, highlighting that partial lymph nodal involvement must be considered as a possible cause of misclassification (Figure 1).

**Figure 2:**
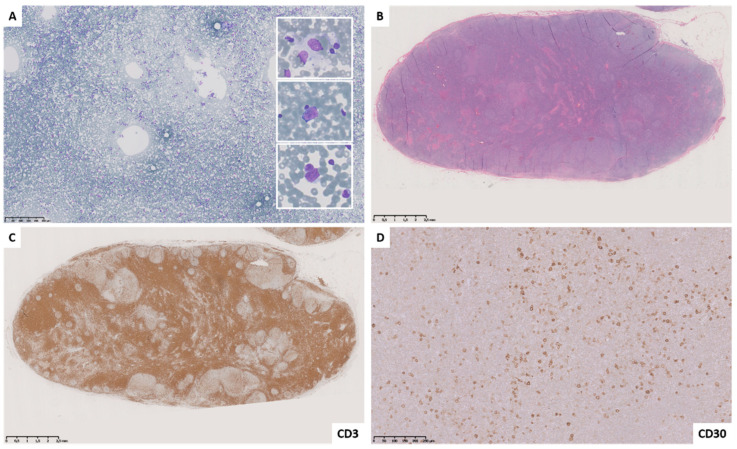
Case L3–4. M, 47 years and history of T-cell rich B-cell lymphoma. Fine needle cytology smear shows a dispersed polymorphous cell population represented by small lymphocytes and few large cells with enlarged and slightly irregular nuclei and scant cytoplasm ((**A**) Diff–Quik stain). Histology revealed a benign reactive hyperplasia ((**B**) hematoxylin and eosin stain) with interfollicular expansion ((**C**) CD3) and numerous CD30-reactive immunoblasts (**D**).

**Figure 3:**
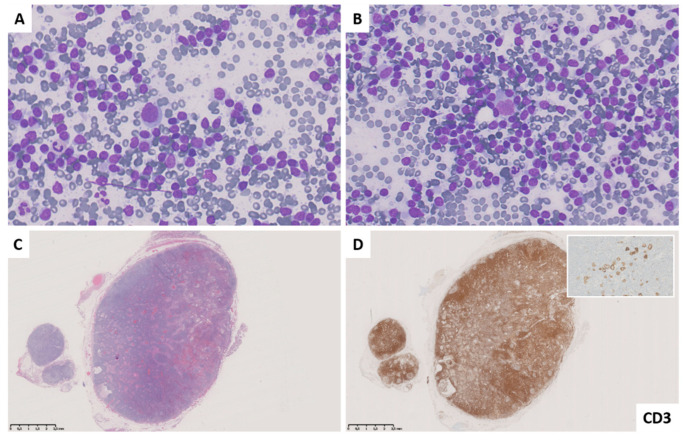
Case L3–5. M, 31 years and history of Hodgkin lymphoma. Fine needle cytology smear shows a dispersed polymorphous cell population represented by small to medium sized lymphocytes and few large cells with enlarged and slightly irregular nuclei ((**A**,**B**), Diff-Quik stain). Histology revealed a benign reactive hyperplasia ((**C**), hematoxylin and eosin stain) with interfollicular expansion ((**D**), CD3) and numerous CD30-reactive immunoblasts (inset).

As well as in other cytological reporting systems, the introduction of an “indeterminate” category in the classification of lymph nodes FNCs aims to maintain a high negative and positive predictive value in the L2 and L5 categories, respectively. Therefore, the L3 category represents an heterogenous group of entities that, in our experience, showed an intermediate ROM (58.3%). As far as diagnostic accuracy is concerned, the higher number of discordant cases (n = 5) was observed just in this category; interestingly, in all these cases, an L3 diagnosis was rendered based on the presence of an excess of large cells with enlarged and slightly irregular nuclei, prominent nucleoli, and scant cytoplasm, whereas histology revealed BRH, most with interfollicular expansion (Table 6, Figure 2 and Figure 3). Although the FC analysis showed non-neoplastic cells and, in two cases (L3–1 and L3–4) a prevalence of T-cell component suggesting the possibility of interfollicular expansion, the evidence of large cells in patients with history of lymphoproliferative disorders was considered of uncertain significance. Notably, despite ancillary techniques were non-contributory in three cases (FC in case L3–2, ICC in cases L3–3, and L3–5), FNC repetition was not performed, and excision biopsy was requested, probably based on clinical history or suspicion.

Finally, although the same ROM (100%) was observed in categories L5 and L4, it is possible that this latter represents an overestimation related to the small number of available histological controls (n = 9). However, we can safely assume that management recommendations in L4, including FNC repetition with acquisition of additional material for ancillary techniques or core-needle/excision biopsy, is substantiated by a highly expected ROM value. A “second-line” approach may be considered to be the more valuable use of core needle biopsy (CNB). In fact, the capability to collect additional material in the L3 and L4 categories represents the major advantages of CNB.

In addition to the basic diagnostic information and the assignment of a diagnostic category, the Sydney system recommends providing, if possible, a second diagnostic level focused on the identification of specific benign or malignant entities. In our experience, the second diagnostic level was provided more frequently in malignant conditions (L4 and L5); in fact, only in nine L2 cases a specific entity was identified, mainly assessing cytological features. Conversely, in malignant settings, the results of ancillary techniques were crucial to provide a second diagnostic level; in particular, specific cluster of differentiation (CD) co-expressions shown by FC, coupled with cytological features and ICC findings, suggested a specific subtype in 26 cases, mostly histologically confirmed (i.e., a small cell population co-expressing CD19/CD10 and CD5- was diagnostic for FL, while CD23/CD5 co-expression was consistent with CLL/SLL; finally, ICC nuclear positivity for cyclin D1 in a CD5+ cell population was diagnostic for MCL). Moreover, the application of ICC panels driven by clinical data and morphological appearance enabled the identification of the site of origin of metastases in 62 cases.

In conclusion, FNC coupled with ancillary techniques is effective in the evaluation of lymphoadenopaties; the implementation of the Sydney system, by the introduction of a standardized categorization, may improve the lymph node FNC diagnostic accuracy. Moreover, clinical practice would benefit from management recommendations specific for diagnostic categories with increasing ROMs, as reported in our experience. The most significant limitations of our study were the single institution and retrospective nature and the low number of cases; therefore, further studies with larger sample sizes are required to confirm the Sydney system’s usefulness.

## Figures and Tables

**Table 1:** Sample characteristics, clinical data, and diagnostic categories of 300 lymph node FNCs.

	No.	%
**Sex**		
Female	173	57.7%
Male	127	42.3%
**Age**		
Mean	54.6	
Minimum	85	
Maximum	13	
**Medical history**		
Previous pathological diagnosis	66	22%
No relevant history	234	78%
**Location**		
Cervical group	136	45.3%
Axillary	55	18.3%
Mandibular	40	13.3%
Inguinal	29	9.7%
Supraclavicular	26	8.7%
Abdominal	8	2.7%
Pectoral	2	0.7%
Iliac obturator	1	0.3%
Pararectal	1	0.3%
Tracheal	1	0.3%
Sternal	1	0.3%
**Ancillary techniques**		
Flow cytometry	109	36.3%
Immunocytochemistry	84	28%
Molecular biology	1	0.3%
None	121	40.3%
**Diagnostic categories**		
L1 Inadequate/non-diagnostic	20	6.7%
L2 Benign	104	34.7%
L3 AUS/ALUS	25	8.3%
L4 Suspicious	13	4.3%
*NHL*	6	2%
*HL*	3	10%
*Metastases*	4	1.3%
L5 Malignant	138	46%
*NHL*	48	16%
*HL*	9	3%
*Metastases*	81	27%

Abbreviations. NHL, non-Hodgkin lymphoma; HL, Hodgkin lymphoma.

**Table 2:** Second diagnostic level and corresponding histological control.

Diagnostic Category	Second Diagnostic Level (n°)	Histological Control (n°)	Histological Diagnosis
**L2**	9	2	Granulomatous lymphadenitis (n = 1),
			dermatopatic lymphadenitis (n = 1)
**L4**	9	7	
*metastases*	4	3	Breast (n = 1)
			PTC (n = 2)
*HL*	3	2	HL (n = 2)
*NHL*	2	2	FL (n = 2)
**L5**	97	71	
*metastases*	62	42	Breast (n = 15),
			PTC (n = 8),
			NSCLC (n = 5),
			squamous cell carcinoma (n = 5),
			melanoma (n = 3),
			colon (n = 1),
			SCLC (n = 1),
			MTC (n = 2),
			seminoma (n = 1),
			NEC (n = 1)
*HL*	9	8	HL (n = 8)
*NHL*	26	21	DLBCL (n = 9),
			FL (n = 7),
			CLL/SLL (n = 3),
			MCL (n = 2)
Total	115	80	

Abbreviations. NHL, non-Hodgkin lymphoma; HL, Hodgkin lymphoma, PTC, papillary thyroid carcinoma; NSCLC, non-small cell lung cancer; SCLC, small cell lung cancer; MTC, medullary thyroid carcinoma; NEC, neuroendocrine carcinoma; DLBCL, diffuse large B cell lymphoma; FL, follicular lymphoma; CLL/SLL, chronic lymphocytic leukaemia/small lymphocytic lymphoma; MCL, mantle cell lymphoma.

**Table 3:** Correlation between Sydney system diagnostic categories and histology/clinical follow-up.

	Clinical Follow-Up n^o^.	Histopathologic Correlation n^o^.	Lost n^o^.	Total
**L1**	0	4	16	20
**L2**	84	20	0	104
**L3**	0	12	13	25
**L4**	2	7	4	13
*LNH*	*1*	*3*	2	
*LH*	*0*	*2*	1	
*Metastases*	*1*	*2*	1	
**L5**	53	60	25	138
*LNH*	*13*	*28*	7	
*LH*	*0*	*8*	1	
*Metastases*	*40*	*24*	17	
**Total**	139	103	58	300

Abbreviations. NHL, non-Hodgkin lymphoma; HL, Hodgkin lymphoma.

**Table 4:** Sensitivity, specificity, positive predictive value, negative predictive value, and accuracy of lymph node FNC.

Statistic	Value	95% CI
Sensitivity	98.47%	94.59% to 99.81%
Specificity	95.33%	89.43% to 98.47%
Positive predictive value	96.27%	91.64% to 98.38%
Negative predictive value	98.08%	92.80% to 99.51%
Accuracy	97.06%	94.03% to 98.81%

**Table 5:** Stratification of ROM in the Sydney system diagnostic categories.

Sydney System Diagnostic Category	Histological or Clinical Follow-Up	Confirmed Malignant Lesions	Risk of Malignancy (ROM)
L1	4	2	50%
L2	104	2	1.92%
L3	12	7	58.3%
L4	9	9	100%
L5	113	113	100%

**Table 6:** Sample characteristics, clinical data, and diagnoses of false positive cases in the present series.

CASE	SEX	AGE	MEDICAL HISTORY	CYTOLOGICAL FEATURES	FC	ICC	HISTOLOGY
L3–1	M	46	Atypical cutaneous lymphoid hyperplasia	Dispersed cell population composed of medium-size lymphocytes with irregular nuclei	Non-neoplastic B and T lymphocytes; prevalence of T-cell component	-	BRH with interfollicular expansion
L3–2	M	27	-	Mature lymphocytes and large nucleolated cells (possibly immunoblasts)	FC non-contributory	-	BRH with interfollicular expansion and large amount of immunoblasts
L3–3	M	66	Mycosis fungoides	Scant cellularity, medium to large size lymphocytes with irregular nuclei	Non-neoplastic B and T lymphocytes	non-contributory: scant CB cellularity	BRH with interfollicular expansion
L3–4	M	47	T-cell rich B-cell lymphoma	Mature lymphocyte and a few medium-to-large sized lymphocytes with irregular nuclei	Non-neoplastic B and T lymphocytes; prevalence of T-cell component	-	BRH with interfollicular expansion and large amount of immunoblasts
L3–5	M	31	Hodgkin lymphoma	Mature lymphocyte, eosinophils, neutrophils, and extremely rare large nucleolated cells	Non-neoplastic B and T lymphocytes	non-contributory: scant CB cellularity	BRH

Abbreviations: FC, flow cytometry; ICC, immunocytochemistry; CB, cell block; BRH, benign reactive hyperplasia.

## Data Availability

Not applicable.
